# Re-Entry Evaluation of Chinese Blood Donors with Unconfirmed Hepatitis B Screening Results

**DOI:** 10.3390/v14112545

**Published:** 2022-11-17

**Authors:** Xuelian Deng, Liang Zang, Daniel Candotti

**Affiliations:** 1 Dalian Blood Center, No. 90 Yan’an Road, Zhongshan District, Dalian 116001, China; 2 Department of Virology, Henri Mondor Hospital, Paris-Est University, Inserm U955-IMRB-Team 18, 94010 Creteil, France

**Keywords:** hepatitis B virus, transfusion, blood donors, follow-up, confirmatory testing, re-entry

## Abstract

The hepatitis B virus (HBV) remains a high priority for Chinese blood banks due to the high prevalence of infection. HBV blood safety has been significantly improved by the implementation of highly sensitive and specific serological and molecular HBV screening assays. The multiplication of viral markers tested and the ever-increasing analytical sensitivity of the tests can make the interpretation of the results difficult. False-positive or indeterminate results may lead to permanent donor deferrals and conflicts between donors and blood banks. To avoid blood shortages, blood services aim to limit unnecessary donor losses by developing procedures for the re-entry of donors temporarily deferred due to an unconfirmed HBV reactivity. The development of such procedures based on donor follow-up and HBV confirmation remains limited. A review of the scarce data available revealed considerable heterogeneity in testing methods and re-entry algorithms, limited validation studies, and a lack of accurate assessment of the residual infectious risk potentially associated with donor re-entry. In conclusion, systematic and widely validated confirmatory testing and prolonged follow-up are essential for safe re-entry of temporary deferred donors. Standardization of HBV testing methods and the establishment of dedicated expert laboratories are needed because of the complexity of HBV infection in blood donors.

Blood screening plays an essential role in reducing the transmission of infectious diseases by transfusion. Viral blood safety has been tremendously improved over the past decades due to the improvement of blood donor selection and screening strategies, the continued development of highly efficient serological and molecular detection assays, and, eventually, the implementation of pathogen reduction technologies in blood components. Viral marker screening of donated blood remains key to reducing transfusion-transmitted infections. However, the use of assays with increasing analytical sensitivity and the growing number of viral markers targeted may lead to an increased risk of false-positive results [1,2]. In the context of a widespread blood shortage, blood donation discard and donor permanent deferral caused by false-positive blood testing results have received redoubled attention [3,4,5]. Confirmation of initially reactive results appears essential to preserve and to maintain a stable blood supply. Identification of true positives is also necessary to accurately monitor the prevalence and incidence of infectious diseases in blood donors, which plays an additional role in blood safety.

The availability of technology and resources may limit the implementation of confirmatory strategies in the blood banks of several countries, including China [6]. Due to the high prevalence of hepatitis B virus (HBV) in the Chinese population, HBV screening of blood donations remains a high priority for blood banks. Improved test sensitivity and the implementation of a screening strategy based on routine detection of HBV surface antigen (HBsAg), using two independent assays and concomitant detection of HBV DNA, have significantly reduced the HBV transfusion–transmission residual risk [7]. However, confirmatory testing is not yet in place at many blood banks, and donors with false-positive results are permanently deferred, resulting in donor loss and legal disputes between donors and blood banks [8]. Here, we will briefly review the history of the HBV screening strategy for blood donors in China and the consequences of the lack of confirmatory testing. Then, the development, implementation, and limitations of donor confirmation and re-entry strategies will be discussed, based primarily on the experience at Dalian Blood Center.

## 1. History of HBV Screening in Chinese Blood Donors

**Figure 1:**
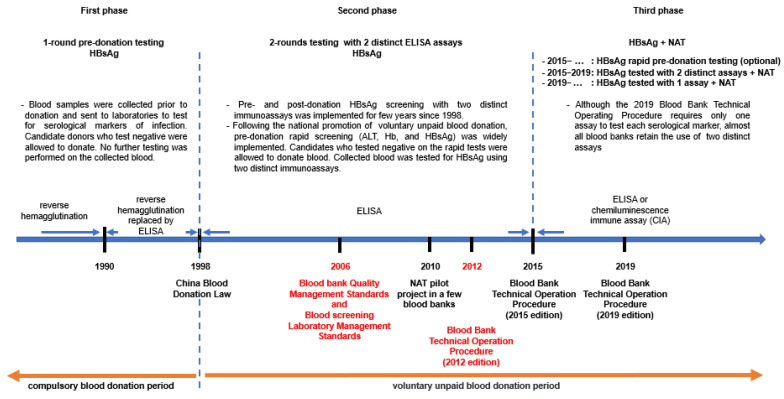
History of HBV screening in Chinese blood donors.

Over the past few decades, China’s blood donor HBV screening strategy has undergone three phases, mainly driven by continuous technological development, including the automation of serological and molecular tests with improved specificity and sensitivity, and the implementation of national standards for blood bank quality management and blood screening laboratory management (Figure 1 and Supplementary Materials) [9,10]. The first phase was the period before the enactment of China’s Blood Donation Law in 1998, the so-called compulsory blood donation period [7]. Candidate blood donors were tested pre-donation for HBV surface antigen (HBsAg) in a dedicated laboratory by using, initially, a reverse hemagglutination method that was progressively replaced by enzymatic immunoassays (EIAs), including enzyme-linked immunoabsorbent assays (ELISAs) between 1990 and 1998. Individuals who tested negative were allowed to donate and no further testing was performed. In the second phase (1998–2015), voluntary unpaid blood donation was promoted nationwide, and a questionnaire on risk behaviors and drug use, a physical examination, pre-donation rapid hemoglobin testing, and dual pre- and post-donation HBsAg testing with distinct immunoassays were implemented through the 1998 Blood Donation Law and Ministerial Order No. 2 of the Ministry of Health. During this period, HBsAg rapid tests largely replaced EIAs for pre-donation screening while two EIAs were still used for post-donation testing. In addition, beginning in 2010, multiplex nucleic acid testing (NAT) technologies for the simultaneous detection of HBV DNA, HCV RNA, and HIV-1 RNA began to be evaluated in a few pilot blood banks around the country. The third phase has been underway since 2015. Between 2015 and 2019, candidate donors were often tested prior to donation with HBsAg rapid tests, and donations collected from eligible donors were tested further for HBsAg with two different EIAs and concomitantly for HBV DNA with multiplex NAT.

Initially, dual HBsAg testing was introduced to compensate for the often-unsatisfactory sensitivity and specificity performance of early generations of domestic assays that could provide false-negative results. Many blood screening laboratories combined a domestic assay with a more sensitive imported assay. In addition, some laboratories defined a gray zone by lowering the reactivity threshold value to limit the risk of non-detection of low-value positive samples. Although the 2019 Blood Bank Technical Operating Procedure recommends that collected blood donations be tested for HBsAg at least once and for HBV, HCV, and HIV-1 nucleic acids, the majority of blood banks still use two EIAs for HBsAg testing. The introduction of NAT either in minipools of plasma samples (MP-NAT) or in individual donation (ID-NAT) significantly reduced further the HBV transfusion–transmission residual risk by improving the detection of HBV primo-infection during the serological window period (WP) and by detecting HBsAg-negative occult hepatitis B virus infection (OBI) [11,12].

## 2. Challenges of False Reactive Results in Blood Donation Screening

Transfusion safety in relation to infectious diseases remains an extremely sensitive and emotionally charged topic in the general public consciousness and particularly among all blood transfusion stakeholders. Consequently, any positive screening result, even if doubtful, leads to the decision to discard the donation and to permanently defer the donor in order to take no risk and to ensure optimal transfusion safety. However, this dogma is increasingly challenged by the multiplication of viral serological and molecular markers tested, especially for HBV, and the number and high sensitivity of recent assays used. Consequently, the final interpretation of a positive screening result can be particularly difficult, and blood screening laboratory staff must often face the question: is an isolated reactive result a true or false positive result?

During the period of compulsory blood donation, blood banks and the public had little awareness of false positives and did not really question the test results. However, the introduction of dual HBsAg testing resulted in cases of samples with discrepant serology results. The number of donations discarded and donors permanently deferred based on a single-assay reactivity became a growing issue for blood banks. Confirmation of seropositivity was hampered by the lack of available matching antibody neutralizing reagents in almost all commercial HBsAg EIA kits, and re-testing with the same assay was not satisfactory. Additional testing for antibodies to the HBV core protein (anti-HBc) was of little value in a high-endemic country such as China, except if anti-HBc seroconversion was observed in follow-up, providing support to maintain dual HBsAg testing [13,14].

The implementation of NAT provided an indirect means to confirm HBsAg reactivity, although several studies reported that no HBV DNA was detected in the majority of samples with HBsAg discrepant results [15,16]. However, discrepancies between HBsAg and HBV DNA testing (HBsAg NR/HBV DNA R) raised additional concerns. The extremely high sensitivity of manufactured NAT assays (Table 1) made it sensitive to cross-contaminations and required special attention from the staff and adherence to very strict new operating rules. In the absence of detectable serological markers (i.e., WP), false-positive NAT results due to cross-contamination may be ruled out by re-testing a clean sample from the initial plasma bag and by donor follow-up. In addition, while the cobas TaqScreen MPX v2 and other real-time PCR-based assays allow the simultaneous detection and direct identification of HBV, HCV, and HIV by using virus-specific probes labeled with different dyes, the cobas TaqScreen MPX v1 and Procleix Ultrio Plus assays indicate the presence of viral genomes with a single consensual signal that does not discriminate between these viruses. Therefore, three additional separate virus-specific discriminatory NAT assays are necessary to identify the virus in the originally reactive sample. Donations tested reactive in the initial multiplex assay might be non-reactive in the discriminatory assays (NDR) and/or in the multiplex assay when repeated (NRR). These discrepancies probably reflect Poisson distribution statistics of HBV DNA levels around the assay’s limit of detection (LoD), especially in OBI donors characterized by extremely low HBV DNA load in plasmas. Confirmation of NDR or NRR samples can be achieved through multiple repeat tests or through the use of enhanced alternative in-house NAT procedures associated with donor follow-up that, unfortunately, may be applicable only in few blood banks [14,17].

Historically, Chinese blood banks have not been responsible for confirming blood-screening reactive results. According to current regulations, only samples that react to HIV serology and/or HIV RNA should be sent to local Centers for Disease Control and Prevention to confirm HIV infections. This lack of confirmatory testing in blood banks has several negative effects. First, it is difficult for donors to understand the meaning of unconfirmed testing results. They usually turn to the hospitals for a new test. However, the usually inconsistent results between hospitals and blood banks due to difference in the analytical sensitivity of the assays used in these different settings, make blood donors even more confused, anxious, and upset. This may lead to conflicts and disputes between blood banks and donors, which can even damage the credibility of blood banks and have a negative impact on the recruitment of new volunteer blood donors. Second, permanent deferral of blood donors results in a significant reduction of the donor pool. A national survey on blood screening in China showed that donations with discrepant screening results accounted for 40% of unqualified donations between 2009 and 2011, and more than 800,000 donors were permanently deferred due to discrepant screening results over the past 10 years [18]. NAT NDR or NRR permanently deferred donors represented 50–60% of NAT-reactive donors [11,19]. According to blood services feedback, a significant number of candidate donors with unconfirmed infection status are requesting the right to continue donating blood (unpublished data). A robust and accurate confirmatory testing strategy is also needed to clearly identify false-positive individuals before safe re-entry into the blood donor pool can be considered. Finally, the lack of confirmation to distinguish true and false positive results may introduce biases in blood bank baseline data to accurately compare, evaluate, and select screening reagents and methods and thus impact future quality improvement of blood screening.

## 3. Strategies Developed by Chinese Blood Services to Confirm Blood Screening Results and Donor Re-Entry

Currently, there are no clear rules or strategies at the national level to systematically confirm the reactive results of blood screening in China, regardless of the viral markers tested. At best, some confirmation strategies have been evaluated or are being developed at the local level, with the main objective of identifying suspected false-positive results on an occasional basis to allow re-entry of potential donors. As indicated above, the main purposes of such re-entry strategies remain to reduce the loss of blood donors, alleviate blood supply tension, and resolve disputes between blood banks and donors [20]. These objectives are of major interest especially to medium and small blood banks. However, less than 10% of blood banks across the whole country are operating donor re-entry procedures using a variety of non-standardized strategies, the three most elaborate of which are described below.

**Figure 2:**
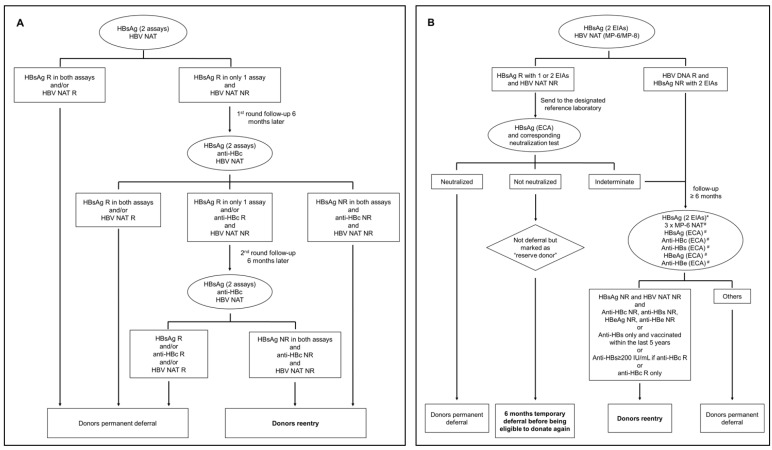

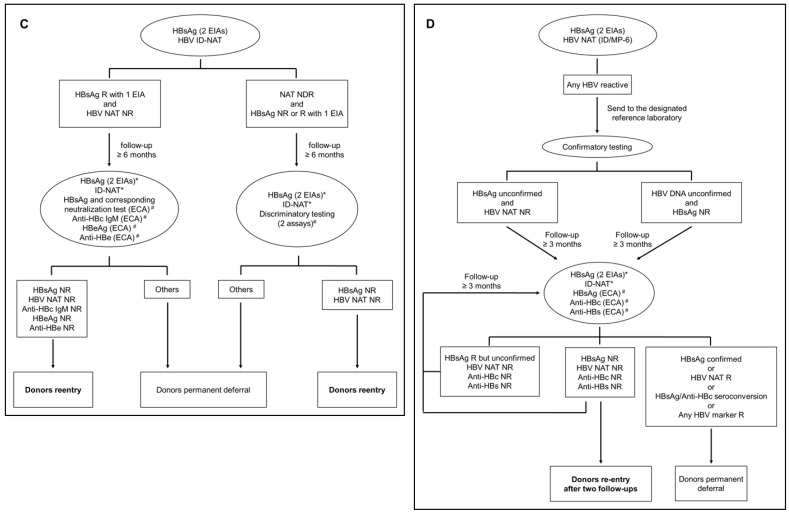
Guidelines for re-entry of donors reactive in a single HBsAg assay and/or NAT nonreactive: CSBT Guidelines (**A**), Jiangsu Guidelines (**B**), Zhejiang Guidelines (**C**), and Dalian Guidelines (**D**). * Assays performed in the blood screening laboratory of local blood banks; ^#^ ECAs and NAT performed in designed reference laboratories.; R, reactive; NR, nonreactive.

First, the Guidelines for Re-entry of Blood Donors with Reactive Blood Screening Test (hereinafter referred to as CSBT Guidelines) and revised versions were issued by the Chinese Blood Transfusion Society. CSBT Guidelines provide a relatively simple guidance for re-entry of only blood donors who are reactive to a single serologic test and NAT non-reactive (Figure 2A) [21]. It does not require confirmatory testing for HBsAg reactive results but recommends that reagents used in follow-up testing have a sensitivity no less than that used for initial blood screening (Table 1). Detection of anti-HBc is the only additional test implemented during follow-up and should identify past HBV exposure and OBI. Follow-up testing is performed at least six months after the last donation. This 6-month waiting period is considered sufficient to warrant anti-HBc seroconversion in cases of early WP HBV infection. If HBsAg, anti-HBc, and HBV DNA are non-reactive at follow-up, donors will be considered eligible for re-entry but still must wait three months to donate blood again (Figure 2A). However, in practice, some blood banks do not strictly adhere to these rules, making modifications based on local screening conditions and their own understanding or interpretation. The main variations are in the HBV screening strategies used in follow-up. For example, most will simply retest a follow-up sample with the same procedures used in the initial screening, while others will use a third HBsAg test with or without anti-HBc screening, or not test for anti-HBc at all, and so on. Ultimately, the vast majority of blood banks apply the rule that a donor is eligible for re-entry only if all HBV markers are negative at the six-month follow-up or beyond. However, some blood banks may also include donors with HBsAg values in the gray area during follow-up testing.

Donor re-entry guidance had also been developed regionally, such as the guidelines issued by the Jiangsu Province Society of Blood Transfusion and the Zhejiang Province Society of Blood Transfusion (hereinafter referred to as the Jiangsu Guidelines and the Zhejiang Guidelines). Blood banks in these two provinces shall re-admit blood donors in accordance with their respective guidelines which are more complex than the CSBT Guidelines (Figure 2B,C). The operability of these guidelines and the effective management of the evaluation criteria and processes are facilitated by the fact that the blood banks in these provinces use fundamentally similar screening platforms and share the same information management system. According to the Jiangsu Guidelines [22], blood banks are responsible for routine blood donation screening and follow-up sample collection if needed. Confirmatory and follow-up testing is performed by the Jiangsu Provincial Blood Center (Figure 2B). HBsAg is confirmed by retesting with an ECA assay and the corresponding neutralization assay. It is not required to confirm HBV DNA reactivity. Then, HBV DNA nonreactive donors tested HBsAg reactive but not neutralized are marked “reserve donor” and subject to a 6-months temporary deferral without any further investigation before being allowed to donate again [22]. HBV DNA nonreactive donors tested HBsAg reactive but indeterminate in the neutralization test and HBV DNA yield donors are followed-up for at least six months. Follow-up samples are tested three times with MP-NAT to reduce the risk of OBI re-entry [23]. In contrast, the Zhejiang Guidelines do not require confirmatory testing prior to follow-up for donors with discordant HBsAg or NAT NDR results at the time of donation [24]. Follow-up samples collected from each blood bank should be sent to designated reference laboratories. Serological confirmation involves re-testing of follow-up samples with the routine HBV screening assays and additional serological tests (Figure 2C). Molecular confirmation in NAT NDR samples is done by testing twice with two different discriminatory NAT assays [24].

Unlike the CSBT Guidelines, both regional guidelines include conditional re-entry criteria for blood donors testing HBsAg NR/HBV DNA NR at follow-up. The Jiangsu Guidelines recommend testing for all HBV molecular and serological markers at follow-up [22]. Donors eligible for re-entry are HBsAg and HBV DNA non-reactive associated with either (i) no reactivity for all additional HBV serological markers; (ii) isolated anti-HBs reactivity and having been vaccinated within five years to limit the risk of anti-HBs-only OBI with transient viremia [25]; (iii) anti-HBc reactivity with protective anti-HBs level ≥200 IU/L as proposed by the Japanese Red Cross Society [26,27]; or (iv) isolated anti-HBc reactivity based on the assumption that natural HBV infection in adults is most often self-limited. Similarly, the Zhejiang Guidelines allow re-entry of donors who have no evidence of active HBV infection, determined by the absence of anti-HBc IgM, HBeAg, and anti-HBe seroreactivity [24]. Anti-HBc reactivity is not considered at all in NAT NDR donors. However, cases of HBV transmission by transfusion have recently been reported in immunocompetent patients who were transfused with blood components from anti-HBc-only repeat donors and whose OBI had gone undetected for years by the most sensitive serologic and molecular screening assays available [28]. The estimated minimum infectious dose per transfusion was approximately 16 copies (or 3 IU) of HBV DNA. In order to detect such a low level of viral load, NAT assays would have to reach a sensitivity limit of 0.8 copies or 0.15 IU/mL [28].

**Figure 3:**
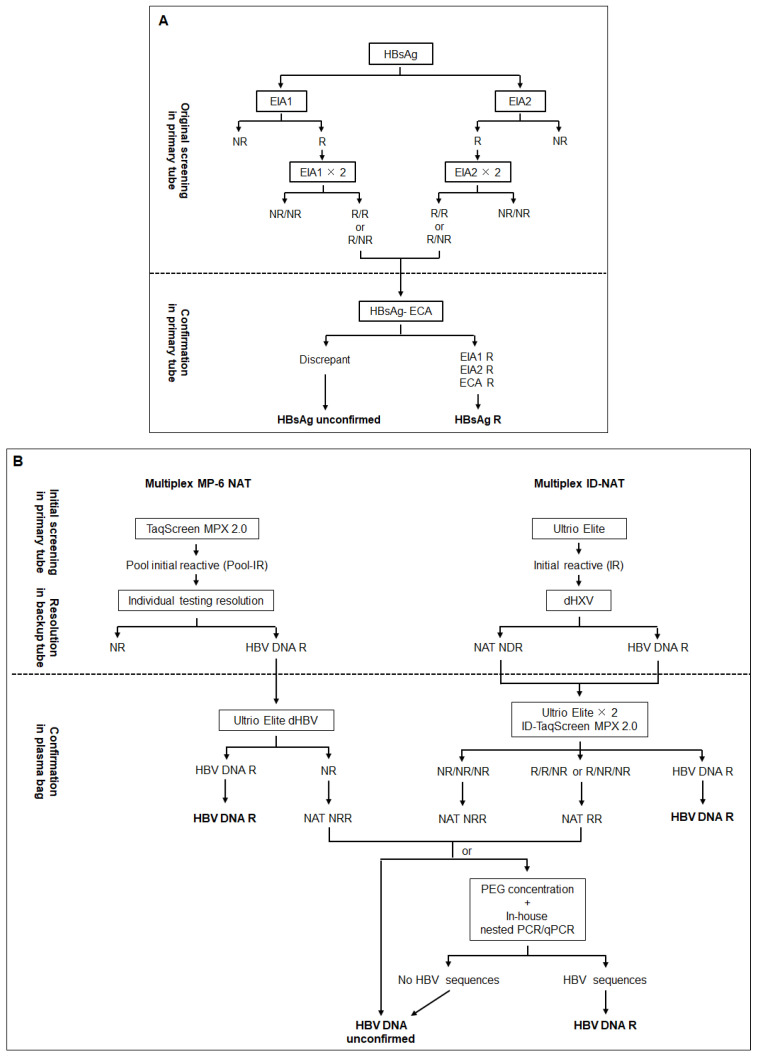
HBsAg (**A**) and HBV NAT (**B**) screening and confirmatory algorithms at Dalian Blood Center.

The Dalian Blood Center has developed and is continuously improving its own confirmatory strategy for HBV blood screening based on the implementation of validated molecular and serological procedures associated with a robust donor follow-up program [8]. This strategy aims to confirm HBV infection in initially reactive donors and to identify those with false-positive test results for re-entry in the donor pool. The first step was to introduce a highly sensitive commercial electro-chemiluminescent assay (ECA) for HBsAg detection and the corresponding neutralization assay as an alternative confirmatory test. However, the neutralization assay failed to clearly confirm HBsAg reactivity in initially weakly reactive samples but that were confirmed HBV DNA positive as reported in other studies (unpublished data), and was eventually dropped. Ultimately, samples reacting with two EIAs and one ECA are considered confirmed HBsAg positive, while samples with inconsistent results are classified as unconfirmed for HBsAg (Figure 3A). Regardless of the NAT assay initially used for screening, samples HBV DNA R or NDR are retested with a different NAT assay or at least twice with the same assay. HBV DNA reactivity is confirmed when detected by two independent assays or detected at least twice by the same assay (Figure 3B). Reactivity with only one type of NAT assay or repeat reactivity with a single NAT assay but NDR classify samples as having unconfirmed HBV DNA. To confirm the presence of very low level of HBV DNA in plasma, putative virions are concentrated from 6 mL plasma by polyethylene glycol (PEG) precipitation, and HBV DNA is detected with extensively validated real-time quantitative PCR and in-house nested PCRs (95% limit of detection: 5–25 IU/mL) [14]. Amplified products are sequenced for definitive confirmation. Donors who are unconfirmed for HBsAg and/or HBV DNA may enter the donor follow-up program for the purpose of potential re-entry (Figure 2D). Donors are then tested for HBV DNA, HBsAg, anti-HBc, and anti-HBs at inclusion and at two consecutive 3-months follow-up. HBV infection is confirmed when HBV DNA is detected, or seroconversion is observed during follow-up, and the donor is permanently deferred. Donors with unconfirmed HBV DNA and whose only serological marker is anti-HBs are not considered eligible for re-entry. Indeed, a recent study showed that 9.5% of Dalian blood donors carrying OBI had isolated anti-HBs (median 62 IU/L; range: 14–914 IU/L) associated with transiently detectable viremia [25]. The HBV transfusion–transmission risk associated with this rare condition remains unknown. Conversely, when HBsAg, HBV DNA, anti-HBc, and anti-HBs antibodies remain continuously nonreactive, they are considered for re-entry and are advised to receive a full vaccination or booster before the next donation.

### 3.1. Blood Donor Re-Entry Using Different Strategies

#### 3.1.1. Donors HBsAg Reactive and NAT Non-Reactive

Data on the re-entry rate of blood donors remains scarce in China. However, recent published data was available from nine blood banks/centers where temporarily deferred donors were reassessed at three and/or six months and beyond in some cases (Table 2). Only Anyang, Jiangsu and Dalian carried HBsAg confirmatory testing before follow-up.

Anyang, Shenzhen, and Zhuhai blood banks/centers reported re-entry rates ranging between 85% and 93% in donors who were HBsAg reactive by only a single EIA at index donation without a confirmatory procedure (except HBsAg neutralization procedure in Anyang) and showed no reactivity on retesting with the same assays for both HBsAg and HBV DNA after a 6-months follow-up (Table 2) [29,30,31]. In Nanning, the re-entry rate was lower (74%), likely due to retesting at follow-up with an additional ECA test whose increased sensitivity presumably improved detection of low-level HBsAg [32]. Chongqing Blood Center carried out follow-up re-testing in accordance with the CSBT Guidelines that includes anti-HBc testing, which probably contributed to the reported 76% re-entry rate [33]. Anhui Blood Center added a third HBsAg EIA in the follow-up procedure on top of the CSBT Guidelines. The combination of three HBsAg EIA tests and anti-HBc testing reduced the re-entry rate to 55% [34]. The Jiangsu and Zhejiang guidelines were more complex as they included testing for several serological markers that could further reduce donors’ chance of being eligible for re-entry. However, re-entry rates of 57% [22] and 64% [24] were reported, respectively. These rates were slightly higher than reported in Anhui despite both centers testing for anti-HBc. This may be due to the fact that Jiangsu and Zhejiang centers allowed re-entry of anti-HBc+ donors who met certain conditions (see above). Dalian Blood Center had the most restrictive conditions that required HBV DNA, HBsAg, anti-HBc, and anti-HBs to be nonreactive. As a result, only 24% of the blood donors followed-up were considered eligible to re-entry (Table 2).

As mentioned above, the Jiangsu Guidelines established a “reserve donor” status for donors HBV DNA nonreactive and HBsAg initially reactive but not neutralized, and who can donate blood according to the standard procedure after a 6-month deferral without follow-up investigation. This may allow donors with HBsAg false-reactivity to return to giving blood as soon as possible, which is beneficial for replenishing the blood supply, reducing the number of donors to be followed-up, and the cost of follow-up. However, it is possible that these “reserve donors” may be reactive with a single HBsAg EIA on the next donation, therefore resulting in the costly discarding of donations once again. Yang D and coauthors reported persistent biological false-positive results in 19% of HBsAg initially reactive blood donors during follow-up [34]. In Dalian Blood Center, continuous or intermittent false HBsAg reactivity was documented in 26% (62/240) of donors showing an isolated HBsAg reactivity at initial screening. Similar observations have been reported by the Australian blood service [35]. Recently, the evaluation of the implementation of the Jiangsu Guidelines showed that 12% of the “reserve donors” were ineligible on their next donation, which was significantly higher than the 5% ineligible rate observed for re-entry donors [22]. Therefore, the authors recommended that the “reserve donor” group be removed and included systematically in the follow-up program to reduce unnecessary donation rejections.

The CSBT Guidelines recommend two 6-month follow-up periods to exclude false-positive HBsAg in donors reactive with a single HBsAg assay. Donors who test HBsAg-reactive twice during follow-up are permanently deferred. A similar approach is used in Dalian Blood Center with the addition of a serological characterization reinforced by the complementary screening of several markers. Extended follow-up and multiplication of markers provides the opportunity to identify both persistent and intermittent false reactivity. This practice has resulted in greater understanding and trust from blood donors in the past and has also relieved pressure from the blood center consultation service [36].

#### 3.1.2. Donors HBV NAT Reactive and HBsAg Non-Reactive (NAT-Yield)

Only a few blood banks/centers reported on investigation about re-entry of donors with unconfirmed NAT reactivity without detectable HBsAg after ≥6-months follow-up (Table 3). No confirmation of the initial HBV DNA test results was considered before follow-up, except in Dalian Blood Center. In these studies, all but two blood centers (Shenzhen and Fujian) tested the follow-up samples using the same assays as for the initial screening plus additional serological and molecular tests, which were repeated in some cases (Table 3). Without testing for additional HBV markers at follow-up, Fujian and Shenzhen reported similar re-entry rates of 76% [37] and 80% [31], respectively, despite significant difference in the numbers and selection of donors followed-up. Henan and Zhejiang Blood Centers introduced supplementary ID-NAT or multiple HBV DNA discriminatory testing and reported lower re-entry rates of 47% [38] and 36% [24], respectively. Usually, discriminatory assays do not fully qualify for confirmation since they are using the same technology and reagents as the initial screening assay. It has been extensively reported that low HBV viremia in blood donations may be undetectable by MP-NAT due to the dilution factor associated with pooling but be detected by more sensitive ID-NAT [39,40,41]. However, a Henan study reported that 47% of samples initially reactive with MP-8 NAT were ID-NAT nonreactive at follow-up [38]. These results may suggest a relatively high level of false-reactive results on initial screening, maybe due to sample cross-contamination. This hypothesis may be supported further by the results from Shenzhen, where only donors with ≥10 donations were selected, probably because false reactivity was intuitively suspected to be higher in regular donors known to be associated with low viral prevalence. Overall, there was no clear relationship between the rate of putative false-reactivity and the type of NAT used (MP-NAT vs. ID-NAT). Furthermore, in the absence of anti-HBc testing, the possibility of OBI with transiently detectable plasma viremia cannot be completely ruled out. Re-entry rates decreased significantly when expanded serological testing was fully associated with HBV DNA detection at follow-up, as observed in Jiangsu (29%), Dalian (24%), and Shanghai (7%) [42] studies. These lower re-entry rates may be due to further disqualification of seropositive donors (i.e., anti-HBc positive) irrespective of the NAT results. While the 7% re-entry rate reported in Shanghai may be related to the relatively small number of cases studied, the 24% rate obtained in Dalian most likely results from the implementation of a systematic and robust molecular confirmation procedure that provides the opportunity to identify falsely NAT reactive samples before inclusion in the follow-up procedure. Therefore, only donors with an unconfirmed HBV DNA reactivity and no HBV serological markers are considered for re-entry and followed-up.

The heterogeneity of re-entry strategies evaluated at different blood banks/centers resulted in significant differences in re-entry rates (7% to 80%) and may call into question their actual impact on blood safety. To estimate the potential residual risk of HBV transfusion–transmission associated with donor re-entry procedures, it is necessary to characterize as accurately as possible the HBV status of donors entering follow-up. However, these data are not available or very limited in most studies. Investigations in Dalian Blood Center can provide some information by combining extensive confirmatory procedures and follow-up at several time intervals of unconfirmed HBV-reactive donors and confirmed NAT yield donors as controls. Between 2010 and 2021, 876 HBsAg-negative donations including 386 (1:1961) HBV DNA R and 490 (1:1545) NAT NDR were screened out of 756,971 donations from 466,911 donors in Dalian Blood Center, of which 497 (57%) were subsequently confirmed HBV DNA reactive (343 HBV DNA R and 154 NAT NDR), 342 (39%) were unconfirmed (30 HBV DNA R and 312 NAT NDR), and 37 (4%) remained unclassified (13 HBV DNA R and 24 NAT NDR) due to lack of available plasma for testing. As part of the confirmatory procedure, extended serologic testing identified 439/497 (88.3%) HBV NAT yield samples as OBI (221 [50.3%] anti-HBc+/anti-HBs−, 176 [40.1%] anti-HBc+/anti-HBs+, and 42 [9.6%] anti-HBc−/anti-HBs+). For the remaining 58 anti-HBc−/anti-HBs− samples, follow-up was needed to distinguish between WP infection, seronegative OBI, or, eventually, DNA false-reactivity. Therefore, donors with confirmed and unconfirmed HBV DNA yields were eligible for follow-up, the main objective of which was to further characterize HBV infection in the former group and to assess the possibility of safe re-entry in the latter.

**Figure 4:**
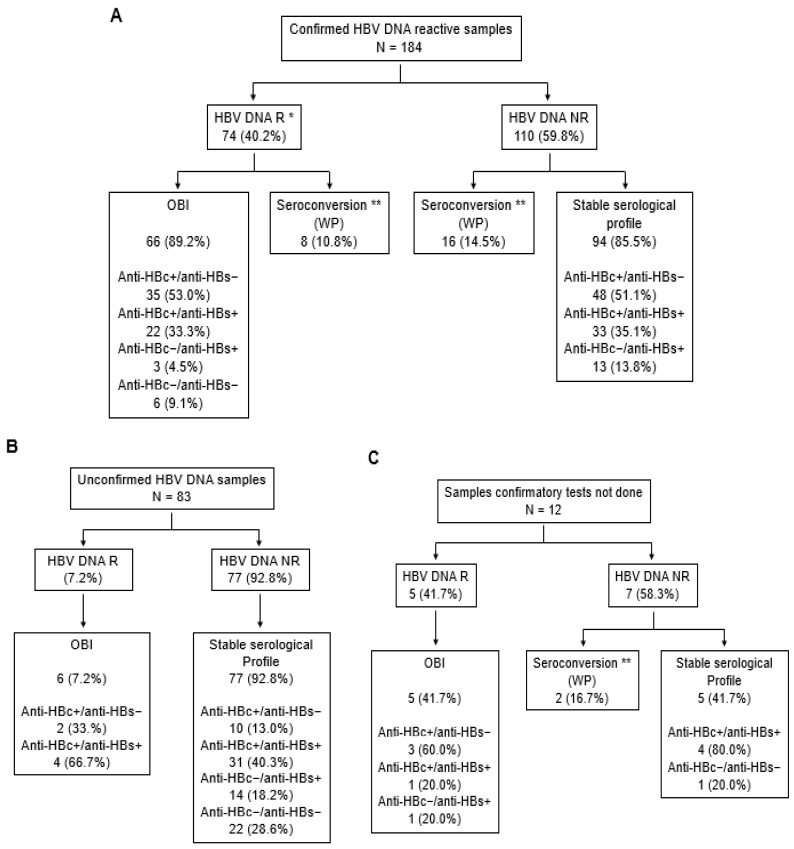
HBV follow-up testing of donors confirmed (**A**) and unconfirmed (**B**) HBV DNA reactive, and donors not tested for confirmation (**C**). * including 31 donors with transiently detectable HBV DNA; ** WP identified by evidence of HBsAg and/or anti-HBc seroconversion.

Two hundred and seventy-nine (32%) of the 876 donors screened HBsAg non-reactive and initially HBV DNA reactive volunteered to participate, of whom 117 (42%) were assessed at two to six time points during follow-up. The remaining donors returned only once. One hundred and eighty-four followed-up donors had confirmed HBV DNA reactivity at index time. HBV DNA was detected in 74 (40.2%) of these donors during follow-up (Figure 4A). However, HBV DNA could only be detected transiently over time, even by ID-NAT, in 31 (42.5%) samples, including 16 donors who showed no reactivity to HBV DNA in the first follow-up sample. Extended serological characterization identified 66 (89.2%) OBIs and eight (11.0%) WP (Figure 4A). In contrast, no HBV DNA reactivity was further observed in 110 (59.8%) samples despite HBV DNA reactivity being previously confirmed by the characterization of partial or complete viral sequences in these samples. Phylogenetic analysis of the 400–3200 nucleotide-long sequences obtained showed no evidence of sample cross-contamination in the multi-step confirmatory procedure (data not shown). Furthermore, anti-HBc reactivity suggested HBV exposure in 81 (73.0%) of these donors, acute infection was documented in 16 (14.4%) others, and 13 (11.7%) showed isolated anti-HBs reactivity. Out of 83 donors with unconfirmed HBV DNA at index donation time, 6 (7.2%) had detectable HBV viremia and could be classified as OBIs (Figure 4B). Among the 77 (92.8%) samples with no detectable viral DNA, 41 were anti-HBc reactive with or without anti-HBs (53.2%), and 14 (18.2%) had isolated anti-HBs. No detectable HBV infection markers were observed in 22 (28.6%) samples. Five (41.7%) and two (16.7%) of the 12 samples lacking confirmatory testing were identified as OBI carriers (HBV DNA+/anti-HBc+ and/or anti-HBs+) and acute WP infection, respectively (Figure 4C). Four (33.3%) HBV DNA-negative samples carried anti-HBc and anti-HBs, and one (8.3%) sample had no HBV marker.

The overall data confirmed that the HBV DNA load is extremely low in OBI carriers and may result in transient NAT reactivity. This substantiates the preferential use of ID-NAT rather than MP-NAT for both screening, confirmation, and follow-up strategies [17]. In addition, testing at several follow-up time points appears to be recommended to limit the risk of non-detection of donors carrying low-level HBV infection. In addition, the implementation of a routine HBV DNA confirmation step at the time viral DNA is first detectable reduces the risk that true OBI carriers will enter follow-up with barely detectable fluctuating viremia and the possibility of being mistakenly considered eligible for re-entry. Anti-HBc-reactive donors who repeatedly tested HBsAg- and HBV ID-NAT-negative with the most sensitive assays available have been reported to be associated with HBV transfusion–transmission [28,43]. This unusual HBV infection pattern could be suspected in 85.3% (81/95) and 53.2% (41/77) of confirmed and unconfirmed HBV DNA-reactive donors showing anti-HBc reactivity in the absence of detectable viremia at follow-up, respectively. Anti-HBc testing also allows identification of early acute infections in follow-up that represent only a minority of cases (9.3% [26/279]). In addition, anti-HBs were detected in 64 of 126 (50.8%) donors who were HBV DNA negative/anti-HBc positive at follow-up. Blood products containing low levels of HBV DNA were found poorly infectious when transfused in the presence of anti-HBs and donations anti-HBc-reactive only that contain anti-HBs levels ≥200 IU/L are considered eligible in some countries [44,45]. However, the protective level of anti-HBs remains a matter of debates as cases of HBV transfusion–transmission despite concomitant detectable anti-HBs have been documented [41,45,46]. In addition, simultaneous detection of HBV DNA and anti-HBs in the absence of detectable anti-HBc was observed in 5.2% (4/77) of OBI donors at follow-up. Isolated anti-HBs were also detected in 13.7% (13/95) and 18.2% (14/77) of HBV DNA confirmed and unconfirmed donors, non viremic at follow-up, respectively. Similarly, an isolated anti-HBs profile has been previously reported in 6.3–9.5% of Chinese blood donors with confirmed OBI and was mainly associated with transient HBV DNA [25,47]. However, a subset of individuals still experienced low but persistent viral replication whose potential for transfusion–transmission remains uncertain [25].

## 4. Issues and Perspectives of Chinese Blood Donor Re-Entry

Anti-HBc testing can add another layer to blood safety by identifying OBI with undetectable viremia. However, there are limitations to anti-HBc screening of Chinese blood donors. Anti-HBc screening does not identify WP infections and the specificity of anti-HBc testing is not optimal, with reported false-reactivity rates of 16–75% according to assays and screening algorithms [17]. The introduction of an additional test, the results of which will be difficult to confirm, in the follow-up of donors for re-entry will inevitably add new difficulties to counselling services and may increase the risk of dispute between donors and blood banks. Nevertheless, the main limitation remains the 30–40% seroprevalence reported in the general Chinese population, which justifies the absence of routine anti-HBc screening in donations [48,49]. Similarly, adding anti-HBc testing to blood donor re-entry strategy is expected to significantly decrease the re-entry rate (Table 2 and Table 3). Therefore, most blood banks seem reluctant to test anti-HBc for donor re-entry as they seek to limit donor loss to relieve pressure on the blood supply. Consequently, there is a risk that some blood banks may be tempted to compromise and adjust their strategy to increase re-entry rate.

Contacting and convincing deferred donors to participate in follow-up remained a critical issue. Dalian Blood Center reported previously a 16% participation rate when donors were contacted between 2013 and 2018 [8]. Three years later, the participation rate increased to 32%, likely due to continuous efforts in educating and training dedicated staff. Getting donors to understand the benefit to them and how it could improve blood safety remains a key factor in promoting donor willingness to participate. In addition, complex re-entry algorithms add organizational and economic constraints to blood services. Decisive limiting factors include the lack of experienced and competent counsellors and the fact that the assessment of donors for re-entry is not yet a legal part of the blood banks’ duties. Domestic blood banks operate entirely on local financial allowances. The cost of implementing re-entry procedures, including labor, testing, construction of the information platform, expensive technical investments, will consume a significant portion of the blood banks’ operating funds. This is particularly true in areas with limited local resources. In contrast, blood banks located in areas with higher local financial resources have the opportunity to apply for additional financial support to develop adequate donor assessment strategies to limit residual infectious upon re-entry.

It is absolutely necessary to scientifically and accurately assess the rationale and the risk for blood safety of Chinese blood donor re-entry strategies. Cost effectiveness should also be considered. For that purpose, evaluation and extensive validation of HBV serological and molecular testing methods/strategies, epidemiological studies based on confirmed data, and proper HBV-infection residual risk assessment are still needed. The National Blood Bank Service System Construction and Development Plan (2021–2025) [50], published in 2021, provides the opportunity to address these issues by establishing a national blood safety monitoring and risk early warning procedure and monitoring the prevalence and residual infectious risk associated with key blood-borne agents. Finally, given the significant regional differences that exist, it would be desirable to establish multiple regional reference laboratories under the management of the National Center for Clinical Laboratories (NCCL, Beijing, China) to promote the implementation of uniform standards for screening and confirmation strategies.

Blood banks around the world are faced with the problem of false-positive HBV test results and their impact on maintaining the pool of safe potential donors. The purpose of this review is to offer readers in the transfusion field some insights into how to deal with this problem based on an overview of the various, albeit imperfect, strategies that have recently been developed in a limited number of Chinese blood banks/centers. It aims to stimulate further reflection and discussion among transfusion stakeholders, which may lead to solutions adapted to different epidemiological and infrastructural contexts.

## Figures and Tables

**Table 1:** Manufactured HBsAg and multiplex NAT assays used for blood donation screening.

HBsAg			NAT		
Assays (Manufacturer)	Analytical Sensitivity (IU/mL)	ID Code *	Assays (Manufacturer)	HBV DNA 95% LoD (IU/mL)	ID Code *
Monolisa HBs Ag ULTRA (Bio-Rad Laboratories, Raymond Poincaré, Marnes-la-Coquette, France)	<0.05	A	Cobas TaqScreen MPX Test		G
			V1.0	3.8	
			V2.0	2.3	
			(Roche Diagnostics, Basel, Switzerland)		
Murex HBsAg Version 3 (Dia Sorin S.p.A., Saluggia, Italy)	0.13	B	PROCLEIX ULTRIO Plus/Elite (Grifols, Barcelona, Spain)	3.4	H
Diagnostic Kit for Hepatitis B Virus Surface Antigen (ELISA) (Livzon Pharmaceutical Group, Zhuhai, China)	≤0.2	C	Nucleic Acid Test Kit for HBV, HCV, HIV(Type-1) (Shanghai Kehua Bio-Engineering Co., Shanghai, China)	≤2.5	I
Diagnostic Kit for Hepatitis B Virus Surface Antigen (ELISA) (Shanghai Kehua Bio-Engineering Co., Shanghai, China)	≤0.2	D	Nucleic Acid Test Kit for HBV, HCV, HIV (Type-1) (DaAnGene Co., Guangzhou, China)	≤100	J
Diagnostic Kit for Hepatitis B Virus Surface Antigen (ELISA) (InTec PRODUCTS, Xiamen, China)	≤0.2	E	Hepatitis B Virus/Hepatitis C Virus/Human Immunodeficiency Virus (1 + 2) (Suzhou Bacme Biotech Co., Suzhou, China)	≤4.2	K
AiD HBsAg ELISA (Beijing WANTAI Biological Pharmacy Enterprise Co., Beijing, China)	0.069	F			

* Identification code of the assays used in testing algorithms discussed by the present study.

**Table 2:** Re-entry of blood donors HBsAg reactive and NAT nonreactive.

Blood Bank/Center	Period	Blood Screening		HBsAg at Index	Confirmatory Testing	Follow-Up Interval	Testing at Follow-Up	Donors Followed Up (N)	Qualified Donors ^†^ (N)	Re-Entry Rate	Ref.
		HBsAg	HBV DNA								
Zhuhai	2017–2019	2 EIAs (C/E)	MP-8 NAT (I)	R or grey area in 1 EIA	No	1× ≥ 6 months	Screening assays *	78	66	85%	[29]
Anyang	2012–2014	2 EIAs (A/F)	ID-NAT (J)	1 EIA R	HBsAg confirmatory V3(Abbott)	1× ≥ 6 months	Screening assays *	53	47	89%	[30]
Shenzhen	2008–2017	2 EIAs (B/D/E)	ID-NAT (H) MP-8 NAT (I)	1 or 2 EIAs R	No	2× ≥ 3 months	Screening assays *	67 **	61	93%	[31]
Nanning	2015–2019	2 EIAs (D/E)	MP-6 NAT (G) MP-8 NAT (I)	R or grey area in 1 EIA	No	1× ≥ 6 months	Screening assays * HBsAg ECA	382	281	74%	[32]
Chongqing	2017–2019	2 EIAs (NA)	MP-6 NAT (G) ID-NAT (H)	1 EIA R	No	1× ≥ 6 months	Screening assays * Anti-HBc (NA)	265	202	76%	[33]
Anhui	2013–2016	2 EIAs (A/F)	ID-NAT (H)	1 EIA R	No	1× ≥ 6 months	Screening assays * HBsAg EIA (C) Anti-HBc EIA	109	60	55%	[34]
Zhejiang	2017–2018	2 EIAs (NA)	ID-NAT (H)	1 EIA R	No	1× ≥ 6 months	Screening assays * Anti-HBc IgM ECA HBeAg ECA Anti-HBe ECA	49	32	65%	[24]
Jiangsu	2014–2019	2 EIAs (D/E)	MP-6 NAT (G) MP-8 NAT (I)	1 EIA R	HBsAg (ECA) + neutralization test (ECA)	1× ≥ 6 months	Screening assays * 3× MP-6 NAT HBsAg ECA Anti-HBc ECA Anti-HBs ECA HBeAg ECA Anti-HBe ECA	860	489	57%	[22]
Dalian	2013–2018	2 EIAs (B/E)	MP-6 NAT (G) ID-NAT (H)	1 or 2 EIAs R	HBsAg (ECA)	2× ≥3 months	Screening assays * ID-NAT HBsAg ECA Anti-HBc ECA Anti-HBs ECA	240 ^#^	58	24%	This study

* Serological and molecular assays used for initial screening; ** Only donors who had donated ≥10 times were involved in the study; ^#^ 142 HBsAg unconfirmed donors (59%) had been followed up ≥2 times; ^†^ No HBV marker detected in follow-up sample(s).

**Table 3:** Re-entry of HBV NAT-yield donors.

Blood Bank/Center	Study Period	Blood Screening		Confirmatory Testing	Follow-Up Interval	Testing at Follow-Up	Donors Followed Up (N)	Qualified Donors (N) ^†^	Re-Entry Rate	Ref.
		HBsAg	HBV DNA							
Shenzhen	2008–2017	2 EIAs (B/D/E)	MP-8 NAT (I) ID-NAT (H)	No	2× ≥ 3 months	Screening assays *	39 **	31	80%	[31]
Fujian	2016–2017	2 EIAs (B/E)	ID-NAT (H)	No	1× ≥ 6 months	Screening assays *	233	176	76%	[37]
Henan	2019–2021	2 EIAs (C/F)	MP-8 NAT (K)	No	1× ≥ 6 months	Screening assays * ID-NAT	174	81	47%	[38]
Zhejiang	2017–2018	2 EIAs (NA)	ID-NAT (H)	No	1× ≥ 6 months	Screening assays * 2 HBV DNA discriminatory assays	110	40	36%	[24]
Shanghai	2012–2017	2 EIAs (B/D)	MP-6 NAT (G)	No	1× ≥ 6 months	Screening assays * ID-NAT HBsAg (ECA) Anti-HBc (ECA) Anti-HBs (ECA) HBeAg (ECA) Anti-HBe (ECA)	30	2	7%	[42]
Jiangsu	2014–2019	2 EIAs (D/E)	MP-6 (G) MP-8 NAT (I)	No	1× ≥ 6 months	Screening assays * 3× MP-6 NAT HBsAg (ECA) Anti-HBc (ECA) Anti-HBs (ECA) HBeAg (ECA) Anti-HBe (ECA)	104	30	29%	[22]
Dalian	2010–2021	2 EIAs (B/E)	MP-6 NAT (G) ID-NAT (H)	Alternative assays	1–4× ≥ 3 months	Screening assays * ID-NAT HBsAg (ECA) Anti-HBc (ECA) Anti-HBs (ECA)	95 ^#^	23	24%	This study

* Serological and molecular assays used for initial screening; ** Only donors who had donated ≥10 times were included in the study; ^#^ 42 (44%) donors with unconfirmed HBV DNA reactivity were followed-up ≥2 times; ^†^ No HBV marker detected in follow-up sample(s); NA, not available.

## Data Availability

Not applicable.

## Supplementary Materials

The following supporting information can be downloaded at: https://www.mdpi.com/article/10.3390/v14112545/s1, Table S1: Abbreviations.

## References

[1] Moore M.C., Howell D.R., Barbara J.A. (2007). Donors whose blood reacts falsely positive in transfusion microbiology screening assays need not be lost to transfusion. Transfus. Med..

[2] Busch M.P. (1997). To thy (reactive) donors be true!. Transfusion.

[3] Tynell E., Norda R., Ekermo B., Sanner M., Andersson S., Björkman A. (2007). False-reactive microbiologic screening test results in Swedish blood donors-how big is the problem? A survey among blood centers and deferred donors. Transfusion.

[4] Zou S., Musavi F., Notari E.P., Rios J.A., Trouern-Trend J., Fang C.T. (2008). Donor deferral and resulting donor loss at the American Red Cross Blood Services, 2001 through 2006. Transfusion.

[5] Kiely P., Hoad V.C., Wood E.M. (2018). False positive viral marker results in blood donors and their unintended consequences. Vox Sang..

[6] Candotti D., Tagny-Tayou C., Laperche S. (2021). Challenges in transfusion-transmitted infection screening in Sub-Saharan Africa. Transfus. Clin. Biol..

[7] National Health and Family Planning Commission of the PRC (2017). China’s Report on Blood Safety 2016.

[8] Deng X., Zang L., Wang X., Chen H., Liu J., Gao Y., Xu S., Wang L., Fan Y., Candotti D. (2020). Follow-up program for blood donors with unconfirmed screening results reveals a high false-positive rate in Dalian, China. Transfusion.

[9] Li L., Li K., Yan K., Ou G., Li W., Wang J., Song N., Tian L., Ji X., Chen Y. (2017). The History and Challenges of Blood Donor Screening in China. Transfus. Med. Rev..

[10] Gao D., Li H., Wang K. (2020). The development of a legal framework for blood donation and blood safety in China over 24 years. BMC Health Serv. Res..

[11] Wu D., Wang X., Feng F., Wang D., Hu Y., Yu Y., Huang J., Wang M., Dong J., Wu Y. (2021). Characteristic of HBV nucleic acid amplification testing yields from blood donors in China. BMC Infect. Dis..

[12] Ye X., Yang B., Zhu W., Zheng X., Du P., Zeng J., Li C. (2013). Six-year pilot study on nucleic acid testing for blood donations in China. Transfus. Apher. Sci..

[13] Deng X., Chen H., Wang X., Zang L., Wang D., Gao H., Zou Y., Zhou L., Li L., Liang X. (2016). The role of HBsAg combined with HBV DNA individual detection in reentry of donors deferred because of HBsAg-reactive. Chin. J. Blood Transfus..

[14] Deng X., Guo X., Li T., Laperche S., Zang L., Candotti D. (2022). Alternative hepatitis B virus DNA confirmatory algorithm identified occult hepatitis B virus infection in Chinese blood donors with non-discriminatory nucleic acid testing. Blood Transfus..

[15] Deng X., An W., Liang X., Zang L. (2012). Application effect of serological detection combined with nucleic acid testing of HBV, HCV and HIV in blood screening in Dalian Blood Center. Chin. J. Blood Transfus..

[16] Dong J., Wu Y., Zhu H., Li G., Lv M., Wu D., Li X., Zhu F., Lv H. (2014). A pilot study on screening blood donors with individual-donation nucleic acid testing in China. Blood Transfus..

[17] Candotti D., Laperche S. (2018). Hepatitis B Virus Blood Screening: Need for Reappraisal of Blood Safety Measures?. Front. Med..

[18] Deng X., Zhang L., Gao Y., Liang X., An W. (2012). Blood screening and blood donor deferral in 357 Chinese blood banks. Chin. J. Blood Transfus..

[19] Ye X., Li T., Shao W., Zeng J., Hong W., Lu L., Zhu W., Li C., Li T. (2019). Nearly half of Ultrio plus NAT non-discriminated reactive blood donors were identified as occult HBV infection in South China. BMC Infect. Dis..

[20] Zhou G., Xie Y., Wang X., Ren Y., Zhu Y. (2014). Blood banks have the responsibility for blood donor re-entry. Chin. J. Blood Transfus..

[21] (2019). Guideline for Reentry of Reactive Blood Donors in Blood Screening Test. https://dev.csbtweb.org.cn/uploads/soft/190413/3_0850526131.pdf.

[22] Jiang Z., Wang J., Pu Y., Wang Y., Chen X., Hu W. (2019). Experiences on the retention and reentry of reactive blood donors in Jiangsu Province. Chin. J. Blood Transfus..

[23] Hu W., Jiang Z., Zhu S., Lin H. (2022). Study on Reentry Evaluation Mode for Blood Donors Used to be HBV Reactive in Jiangsu Province. J. Exp. Hematol..

[24] Zhou H., Wang Y., Sang L., Liu J., Meng Z., Hu W. (2019). Application and establishment of the regionally integrated strategy for the re-entry of blood screening reactive donors. Chin. J. Blood Transfus..

[25] Deng X., Guo X., Gu H., Wang D., Laperche S., Allain J.P., Zang L., Candotti D. (2022). Anti-HBc-nonreactive occult hepatitis B infections with HBV genotypes B and C in vaccinated immunocompetent adults. J. Viral Hepat..

[26] Yugi H., Mizui M., Tanaka J., Yoshizawa H. (2006). Hepatitis B virus (HBV) screening strategy to ensure the safety of blood for transfusion through a combination of immunological testing and nucleic acid amplification testing—Japanese experience. J. Clin. Virol..

[27] Satake M., Taira R., Yugi H., Hino S., Kanemitsu K., Ikeda H., Tadokoro K. (2007). Infectivity of blood components with low hepatitis B virus DNA levels identified in a lookback program. Transfusion.

[28] Candotti D., Assennato S.M., Laperche S., Allain J.P., Levicnik-Stezinar S. (2019). Multiple HBV transfusion transmissions from undetected occult infections: Revising the minimal infectious dose. Gut.

[29] Zhang W., Wu R., Shi D., Hu X. (2021). Analysis on tracking results of ELISA single reagent reactivity or grey zone in blood screening for blood donor re-entry. Int. J. Lab. Med..

[30] Wu L. (2015). Study on deferral and re-entry of blood donors reactive in blood screening. J. Clin. Transfus. Lab. Med..

[31] Zheng X., Xu X., Zeng J., Chen Y., Liu H., Xiong W. (2019). Study on re-entry strategy of blood donor who are reactive to HBV and HCV screening in Shenzhen Blood Center by follow-up. Chin. J. Blood Transfus..

[32] Li X., Jiang Y., Li Y., Wei Z., Zhu Q., Zhang L., Pang D. (2021). Analysis of follow-up testing and re-donation of 676 blood donors deferred in Nanning City. Lab. Med. Clin..

[33] Yang D., Li X., Qin W., Dai H., Huang X., Duan H. (2021). Analysis on the return of blood donors in Chongqing. Chongqing Med..

[34] Cheng W., Zhou X., Zhang Y., Lv Q., Cui W., Wang T. (2019). The analysis on feasibility of re-entry of ELISA single reagent unqualified blood donors. Chin. J. Blood Transfus..

[35] Kiely P., Stewart Y., Castro L. (2003). Analysis of voluntary blood donors with biologic false reactivity on chemiluminescent immunoassays and implications for donor management. Transfusion.

[36] Deng X., Zang L., Liu J., Gao Y., Xu S., Wang L., Gong B., Liang X. (2017). Exploration and practice of the blood donor evaluation procedures in Dalian Blood Center, China. Chin. J. Blood Transfus..

[37] Wang L., Lin S., He X., Zan Y., Wang M. (2019). Analysis of NAT-yield blood donors re-entry. Fujian Med..

[38] Chen M., Wang Y., Yan L., Wei Y., Tu T., Zhao L., Lv Y. (2022). Analysis of influencing factors on re-entry of HBV DNA reactive blood donors. Chin. J. Blood Transfus..

[39] Vermeulen M., Coleman C., Mitchel J., Reddy R., van Drimmelen H., Ficket T., Lelie N. (2013). Sensitivity of individual-donation and minipool nucleic acid amplification test options in detecting window period and occult hepatitis B virus infections. Transfusion.

[40] Enjalbert F., Krysztof D.E., Candotti D., Allain J.P., Stramer S.L. (2014). Comparison of seven hepatitis B virus (HBV) nucleic acid testing assays in selected samples with discrepant HBV marker results from United States blood donors. Transfusion.

[41] Stramer S.L., Krysztof D.E., Brodsky J.P., Fickett T.A., Reynolds B., Dodd R.Y., Kleinman S.H. (2013). Comparative analysis of triplex nucleic acid test assays in United States blood donors. Transfusion.

[42] Ren Y., Zhou G., Wang Z., Zhang Y., Zhao Q., Qian K. (2017). The primary study on the methods and feasibility of re-entry of NAT reactive blood donors. Chin. J. Blood Transfus..

[43] Seed C.R., Maloney R., Kiely P., Bell B., Keller A., Pink J., Blood Service Medical Services Lookback Team (2015). Infectivity of blood components from donors with occult hepatitis B infection—Results from an Australian lookback programme. Vox Sang..

[44] Allain J.P., Mihaljevic I., Gonzalez-Fraile M.I., Gubbe K., Holm-Harritshøj L., Garcia J.M., Garcia J.M., Brojer E., Erikstrup C., Saniewski M. (2013). Infectivity of blood products from donors with occult hepatitis B virus infection. Transfusion.

[45] Taira R., Satake M., Momose S., Hino S., Suzuki Y., Murokawa H., Uchida S., Tadokoro K. (2013). Residual risk of transfusion-transmitted hepatitis B virus (HBV) infection caused by blood components derived from donors with occult HBV infection in Japan. Transfusion.

[46] Levicnic-Stezinar S., Rahne-Potokar U., Candotti D., Lelie N., Allain J.P. (2008). Anti-HBs positive occult hepatitis B virus carrier blood infectious in two transfusion recipients. J. Hepatol..

[47] Zhang L., Chang L., Laperche S., Ji H., Zhao J., Jiang X., Wang L., Candotti D. (2019). Occult HBV infection in Chinese blood donors: Role of N-glycosylation mutations and amino acid substitutions in S protein transmembrane domains. Emerg. Microbes Infect..

[48] Liang X., Bi S., Yang W., Wang L., Cui G., Cui F., Zhang Y., Liu J., Gong X., Chen Y. (2009). Epidemiological serosurvey of hepatitis B in China—Declining HBV prevalence due to hepatitis B vaccination. Vaccine.

[49] Cui F., Shen L., Li L., Wang H., Wang F., Bi S., Liu J., Zhang G., Wang F., Zheng H. (2017). Prevention of Chronic Hepatitis B after 3 Decades of Escalating Vaccination Policy, China. Emerg. Infect. Dis..

[50] National Health Commission of PRC The National Blood Bank Service System Construction and Development Plan (2021–2025). https://csbt.org.cn/uploads/soft/211231/3_1908268081.pdf.

