# Supplementary material for: Re-Entry Evaluation of Chinese Blood Donors with Unconfirmed Hepatitis B Screening Results
Source: Viruses. 2022 Nov 17;14(11):2545. doi: 10.3390/v14112545 (PMC9698129; doi:10.3390/v14112545)
Supplement: Supplementary file 1 [file viruses-14-02545-s001.zip › viruses-1992792-supplementary.pdf]

Supplementary Table S1.

Abbreviations	
ECA	Electro-chemiluminescence assay
EIA	Enzymatic immunoassay
ELISA	Enzyme-linked immunoabsorbent assay
ID	Individual
LoD	Limit of detection
MP	Minipool
NAT	Nucleic acid testing
NDR	Reactive in the initial multiplex assay but non-reactive in the discriminatory assays
NR	Non-reactive
NRR	Reactive in the initial multiplex assay but non-reactive when repeated
OBI	Occult hepatitis B virus infection
WP	Window period
